# Expression of *Mst89B* and *CG31287* is Needed for Effective Sperm Storage and Egg Fertilization in *Drosophila*

**DOI:** 10.3390/cells10020289

**Published:** 2021-02-01

**Authors:** Gurman Grewal, Bahar Patlar, Alberto Civetta

**Affiliations:** Department of Biology, University of Winnipeg, Winnipeg, MB R3B 2E9, Canada; grewal-g85@webmail.uwinnipeg.ca (G.G.); b.patlar@uwinnipeg.ca (B.P.)

**Keywords:** gene expression, sperm gene function, reproduction, fecundity, sperm competition, fertilization

## Abstract

In *Drosophila*, male reproductive fitness can be affected by any number of processes, ranging from development of gametes, transfer to and storage of mature sperm within the female sperm storage organs, and utilization of sperm for fertilization. We have previously identified the 89B cytogenetic map position of *D. melanogaster* as a hub for genes that effect male paternity success when disturbed. Here, we used RNA interference to test 11 genes that are highly expressed in the testes and located within the 89B region for their role in sperm competition and male fecundity when their expression is perturbed. Testes-specific knockdown (KD) of *bor* and *CSN5* resulted in complete sterility, whereas KD of *CG31287*, *Manf* and *Mst89B*, showed a breakdown in sperm competitive success when second to mate (P2 < 0.5) and reduced fecundity in single matings. The low fecundity of *Manf* KD is explained by a significant reduction in the amount of mature sperm produced. KD of *Mst89B* and CG31287 does not affect sperm production, sperm transfer into the female bursa or storage within 30 min after mating. Instead, a significant reduction of sperm in female storage is observed 24 h after mating. Egg hatchability 24 h after mating is also drastically reduced for females mated to *Mst89B* or *CG31287* KD males, and this reduction parallels the decrease in fecundity. We show that normal germ-line expression of *Mst89B* and *CG31287* is needed for effective sperm usage and egg fertilization.

## 1. Introduction

Variation in fecundity and fertility influences an organism’s reproductive fitness. The identification of genes contributing to variation in phenotypes, particularly those that impact fitness, is a long-standing question among evolutionary geneticists [1]. In *Drosophila* and other organisms, gene perturbation screens such as loss-of-function alleles or transgenic constructs that modulate gene expression have been used to identify sperm and seminal fluid protein genes that have an impact on different aspects of male fecundity. Perturbation of specific genes can completely or partially block the production of mature sperm [2], drastically decrease the ability of males to store sperm [3,4,5], disturb the retention and release of sperm in/from storage [6,7,8], and even impair sperm viability once in female storage [9,10]. Both sperm and seminal fluid protein genes have been shown to influence male sperm competitive ability [11]. A gene perturbation approach can be fruitful not only in determining the molecular underpinnings of traits such as fecundity and fertility, but together with the implementation of detailed phenotypic tests, can contribute to the much-needed annotation of yet functionally unknown genes within genomes.

In *D. melanogaster* males, mature sperm are stored in a pair of seminal vesicles that are connected to the ejaculatory duct [12]. During copulation, sperm, alongside seminal fluid proteins from the accessory glands, are transferred to the female. The transfer of male seminal fluid proteins triggers a series of conformational changes in the female bursa that help move transferred sperm towards the anterior end of the bursa and closer to the entrance of female sperm storage organs [13,14]. The female sperm storage organs include a long, coil-shaped seminal receptacle, which opens into the common oviduct at the top of the bursa, and two mushroom-shaped spermathecae connected to the bursa by a duct near its anterior end [12,15]. Thousands of sperm are transferred to the female during copulation, however, only approximately 20% of the sperm are stored [12]. The excess sperm within the bursa and the gelatinous mating plug deposited by the male during copulation are expelled one to six hours after the mating, with the majority expelled approximately three hours after mating [16,17]. The stored sperm are used very efficiently for fertilization [7,12,18,19], which allows the female to produce fertile eggs for up to two weeks [12].

Among internally fertilizing organisms, mature sperm face challenges between insemination and fertilization as sperm interacts with the female structural and biochemical environment and possibly undergoes the molecular modifications required for competency [20,21]. In *Drosophila*, the importance of the female environment is highlighted by changes in the bursa shortly after sperm transfer, which helps with positioning sperm for storage [13,14], influence of spermathecal secretory cells on sperm motility [22], modulation of sperm release, and impact on sperm competition by the nerve terminals innervating the female reproductive tract [17,23,24]. Thus, in any gene perturbation assay seeking to determine effects on sperm function, it becomes critical to examine sperm within the female reproductive tract at different time points after insemination.

We have previously identified, using a combination of genetic mapping, bioinformatics tools, and gene disruption of candidate genes, the 89B cytogenetic location as harboring genes of interest in terms of sperm development, non-competitive male fecundity and sperm competitiveness [25,26]. Our prior work used a combination of gene disruptions via P-element insertions and a non-tissue specific *Act5C*-Gal4 driver to knockdown (KD) gene expression via RNAi [26]. Ubiquitous KD of *Mst89B* significantly reduced second male paternity success (P2) without affecting fecundity, but the reduction in average P2 was less than 10% and the KD did not break down second male advantage (i.e., P2 > 0.5) [26]. The use of a combination of P-element insertion mutagenesis and non-tissue-specific KDs poses the question of whether our prior results might have been influenced by ubiquitous rather than tissue-specific gene effects [26]. Here, we use a *bam*-Gal4 driver to specifically KD gene expression in the germline [2,27]. We conduct germline-specific KD of 11 genes with high expression in the testes located in the 89B cytogenetic region (including three previously tested using *Act5C*-Gal4 [26]) to test their role in male fecundity and second male paternity success. We also test the effects of knocking down these genes on sperm production, sperm transfer, sperm storage, egg fertilization, and egg viability. KD of two genes (*CSN5* and *bor*) rendered fully sterile males. KD of *Manf, Mst89B,* and *CG31287* drastically disrupted P2 and male fecundity. We found that males with the KD of *Manf* produced significantly fewer sperm whereas males with the KD of *Mst89B* and *CG31287* produced normal amounts of sperm. Sperm from these two genes’ KDs were normally transferred and stored in the female’s seminal receptacle, but the amount of sperm retained in storage dropped significantly within the first 24 h after mating. This drop in the number of sperm in storage caused a similar drop in egg fertilization over the same period of time.

## 2. Materials and Methods

### 2.1. Gene Selection

Genes were identified using FlyBase (https://flybase.org). First, all the genes located in the hypothesized 89B hotspot were retrieved from FlyBase and were then screened based on their expression pattern using FlyAtlas anatomy microarray and modENCODE anatomy RNA-Seq data. We selected genes categorized as having high expression in the testes in at least one of the two data sources (Table S1).

### 2.2. Fly Stocks and Maintenance

UAS-hairpin lines for candidate genes were purchased from Vienna Drosophila Resource Center (VDRC) and Bloomington Drosophila Stock Center (BDSC) (Table S2). The KK lines from VDRC and the Harvard Transgenic RNAi Project (TRiP) lines from BDSC were specifically chosen because these stocks contain UAS-hairpin sequences inserted at specific locations in the second chromosome. Specific insertion of the UAS-hairpin construct avoids the variable level of hairpin expression associated with P-element-based constructs due to their random insertion in the genome [28]. The *bam*-Gal4 stock was kindly provided by Dr. Geoffrey D. Findlay (College of the Holy Cross, Worcester, MA, USA). Wild-type flies were purchased from Ward’s Science (stock 87w6550) and ebony flies from BDSC (stock 1658).

All flies were reared on cornmeal-molasses-yeast-agar media and maintained in a 12-h light-dark cycle at 23 ± 1 °C. Parental stocks were maintained by allowing males and females to mate for ten days before discarding them. Flies were anesthetized using CO_2_ for collection and sexing. Post-eclosion, females were collected every five hours to ensure virginity. All experimental procedures were performed with *D. melanogaster* females and males, which were aged for three to six days prior to use.

### 2.3. Gene Knockdown

To KD candidate gene expression, males from lines with the UAS-hairpin were crossed with females from the *bam*-Gal4 driver strain. F1 sexually naïve males were collected from these crosses and the male reproductive tract dissected in 1x PBS to verify gene KD. For each candidate gene and wild-type control, three biological samples were prepared with each sample containing reproductive tracts from six males. RNA was isolated from the whole reproductive tract of KD and wild-type control males using the Bio-Rad Aurum Total RNA Mini Kit. Extracted RNA was quantified and checked for purity using a NanoDrop spectrophotometer (ThermoFisher Scientific, Waltham, MA, USA). Complementary DNA (cDNA) was synthesized using an iScript Select cDNA Synthesis Kit (Bio-Rad, Mississauga, ON, Canada). Three 3-fold serial dilutions were prepared before performing quantitative PCR (qPCR) to test primer efficiencies (Table S2). We tested three reference genes, *RpS18*, *eEF1α1*, and *αTub84B*, and found that *RpS18* showed the highest expression and the least variability in expression between samples (Figure S1). As a result, only *RPS18* was used to normalize all expression data. qPCR reactions were carried out using the iQ SYBR Green Supermix Kit and run in a CFX Connect Real-Time PCR Detection System (Bio-Rad, Mississauga, ON, Canada). The expression level of the target gene in each sample was determined by calculating ∆Cq as the Cq of the reference gene (*RpS18*) minus the Cq of the target gene and ΔΔC_q_ as the ∆Cq of the treatment (gene KD) minus the ∆Cq of the control (wild-type).

### 2.4. Fecundity and Sperm Competition

Single-pair matings were performed between KD or control males and wild-type females to assess their ability to father progeny. On Day 0, virgin females were placed in individual vials (vial 1) with either a KD male or a control male. Matings were visually confirmed and the males were removed immediately after mating. On Day 5, mated females were moved to a new vial (vial 2), and on Day 10 the females were discarded. Progeny from vials 1 and 2 were counted from 164 females seven days after F1 eclosion began.

To determine offensive sperm competitive ability, KD or control males were crossed with females that were homozygous for an ebony (e/e) recessive mutation and had already been singly mated to same-aged ebony males. On day 0, 20 virgin e/e females and 30 e/e males were crossed *en masse*. 24 h later (day 1), the males were discarded, and the females were individually transferred to a fresh vial (vial 1) containing fly media supplemented with active dry yeast. An aspirator was used to transfer flies to avoid use of CO_2_, which can increase copulation latency [29]. On day 3, a single wild-type control or a male with a specific gene KD was added to each vial using an aspirator. Vials were inspected every 12 min for up to eight hours to visually confirm that mating had occurred. Females were transferred to a new fresh vial (vial 2) and allowed to oviposit. Five days after second mating (day 8), females were transferred to a fresh vial for an additional seven days (vial 3). Progeny from 141 females over vials 1, 2, and 3 were counted on the 20th day after the beginning of oviposition and scored based on body coloration, with ebony progeny being fathered by the first male and non-ebony progeny being fathered by the second male. The fraction of progeny in vials 2 and 3 sired by the second male to mate was designated as P2.

### 2.5. Sperm Production, Sperm Transfer, Sperm Storage, Egg Hatchability, and Larva Viability

For males where KD of a gene resulted in low fecundity, sperm production, transfer, and storage as well as egg hatchability and viability were determined. For sperm production, the reproductive tract of three- to six-day old naïve males was dissected to isolate one of their seminal vesicles. The sperm heads in the seminal vesicle were stained with 4′,6-Diamidin-2-phenylindol (DAPI) and counted. The sperm were visualized under a Olympus (Shinjuku City, Japan) BX60 fluorescent microscope and an image was taken for each sample. The software ImageJ with the plugin Cell Counter was used to individually score the number of sperm present in the seminal vesicle.

Sperm transfer and sperm storage was evaluated by using singly mated wild-type females with KD or control males. Mating was visually confirmed, and the females were frozen 30 min and 24 h after the end of copulation (AEC). Sperm transfer was determined by dissecting the reproductive tract of females frozen 30 min AEC and counting the number of sperm present in the bursa. For sperm storage in the primary storage organ, the seminal receptacle [16,30,31], females frozen at 30 min and at 24 h AEC were dissected. Sperm heads were stained with DAPI and counted using ImageJ (bursa) as previously described or directly from the dissected samples (seminal receptacle).

Egg hatchability was evaluated by mating virgin wild-type females *en masse* in bottles containing 60 females and 60 KD or control males. The bottles were capped with petri dishes containing agar tinted with grape juice, and the females were left to lay eggs for 24 h. Each cross was duplicated. The following day, the eggs were counted in the petri dishes and the number of larvae scored for two subsequent days to score egg-hatchability. In addition, we transferred groups of 6 to 10 larvae into individual vials and counted the number of pupae (larva to pupa viability) and number of adults (pupa to adult viability) produced in each vial.

### 2.6. Statistical Analysis

Based on the *a priori* hypothesis that RNAi should decrease gene expression, a one-tailed independent *t*-test was used to test reduction in expression caused by KDs relative to wild-type controls. Phenotypic data, except for viability, was analyzed using one-way ANOVA with treatment (wild-type and KDs) as the main factor, followed by a posteriori Scheffe’s test. Viability data were analyzed using chi-square test of proportions and non-parametric statistics. All statistical tests were conducted using SPSS (version 25). To correct for multiple hypothesis testing, *p*-values were FDR corrected.

## 3. Results

We identified a total of 69 protein coding genes located in the 89B region and focused on 13 genes with high testes expression (Table S1). All candidate genes had stocks available from either VDRC or BDSC stock centers, except for *CG42446*. The remaining 12 genes (Table S2) were KD, and gene expression was compared against wild-type controls. KD of *CG5903* did not result in significant down regulation of gene expression (Figure S2). For the remaining 11 genes, the effect of each gene germline down regulation was tested in single mating fecundity and sperm competition assays.

### 3.1. Reduced Germline Expression via RNAi Identifies Genes Needed for Male Fecundity

**Figure 1:**
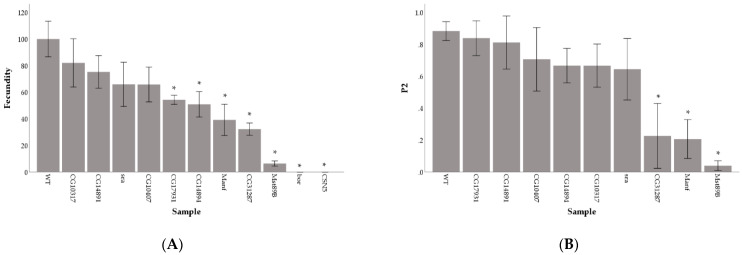
Average fecundity (**A**) and second male paternity success (**B**) for gene knockdowns compared with the wild-type (WT) control. Asterisks (*) above bars identify samples with averages significantly different from the wild-type control. Error bars are 2 standard errors of the mean.

We found significant differences in fecundity among males tested (F_11,152_ = 38.7; *P* < 0.001). A Scheffe’s post-hoc test shows that compared with control wild-type males, germline KD of 7 genes resulted in decreased fecundity (FDR corrected *p*-values, q < 0.01; Figure 1A). In particular, KD of five genes (*Manf*, *CG31287*, *Mst89B*, *CSN5*, *bor*) displayed the strongest effect, with more than 50% reduction in fecundity. Males with the KD of *CSN5* and *bor* were sterile with no adult offspring being produced. In comparison to control males, males with the KD of *Mst89B*, *Manf*, and *CG31287* only fathered 6.5%, 39.2%, and 32.3% of the progeny, respectively (Figure 1A). As expected, sterile males (KD of *CSN5* and *bor*) sired no progeny in sperm competition assays. For the other gene KDs, we found significant variation among tested males in second male paternity success (F_9,131_ = 20.8; *P* < 0.001) and a significant correlation between single-pair mating fecundity and sperm competitive ability (Pearson correlation coefficient r = 0.923; *P* < 0.001; R^2^ = 0.851). Only the KDs of *Mst89B*, *Manf* and *CG31287* had significantly lower second male paternity success than wild-type males (Scheffe’s post-hoc test, q < 0.01), with the KD causing a breakdown of second male paternity success (average P2 < 0.5) (Figure 1B).

**Figure 2:**
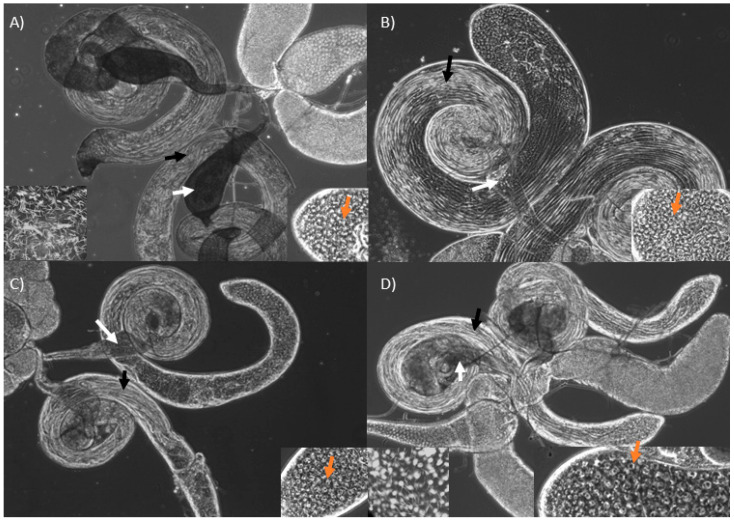
Male reproductive tract of wild-type (**A**) and KDs of *bor* (**B**), *CSN5* (**C**) and *Manf* (**D**). Elongating cyst (black arrows) and seminal vesicles (white arrows) are shown. The seminal vesicle of KD males are small and lighter in color due to complete lack (**B** and **C**) or few mature sperm (**D**). The right bottom panels show presence of spermatocytes at the testes apical end (orange arrows). The left bottom panel (within **A** and **D**) show many and few mature sperm, seen as white needle-like structures, within a section of a wild-type and a *Manf* KD seminal vesicle, respectively.

When compared with wild-type males (Figure 2A), KD of *bor* and *CSN5* have visible spermatids but smaller seminal vesicles devoid of sperm (Figure 2B,C). *Manf* KD males had a similar phenotype, but their seminal vesicles contained some mature sperm (Figure 2D). We found abundant spermatogonia and spermatocytes at the apical end of the testes in all KDs (Figure 2, orange arrows), and the cysts appear to elongate normally to form sperm bundles (Figure 2, black arrows). The clearest difference among these KDs relative to wild-types is the complete lack of mature sperm in KDs of *bor* and *CSN5*, and the very small number of sperm produced by *Manf* KDs (Figure 2).

**Figure 3:**
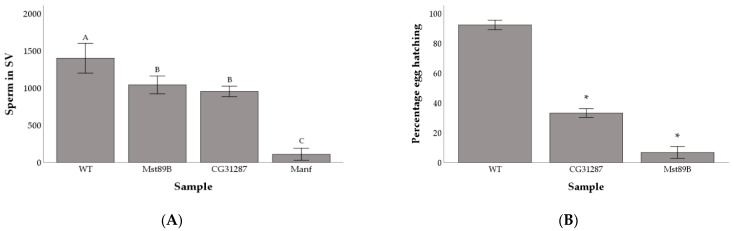
Average number of sperm cells in the male seminal vesicle (SV) (**A**) and percentage of female eggs hatching when mated to control versus gene knockdowns (**B**). Shared letters above bars identify non-significantly different groups. Asterisks (*) above bars denote significant difference between the samples and the wild-type control. Error bars are 2 standard errors of the mean.

We followed up on the fertility of *Mst89B*, *Manf*, and *CG31287* KD lines by quantifying the amount of sperm cells present in the seminal vesicle (Figure S3). We found significant differences in sperm production among wild-type males and the KDs for the three genes (F_3,47_ = 83.01; *P* < 0.001). Males with perturbed expression of *Mst89B* and *CG31287* had significantly fewer sperm in their seminal vesicles than wild-type males (q = 0.004 and q = 0.0001 respectively), but large amounts of sperm could still be detected (Mean ± standard deviation: 1,039 ± 199 and 952 ± 132 respectively). Comparatively, males with the KD of *Manf* produced very few sperm (107 ± 150) (Figure 3A). *Manf* KDs produced, on average, more offspring than KDs of *Mst89B* (Figure 1A) despite producing so few sperm (Figure 2A). While it is possible that *Manf* KDs might be able to boost females’ egg-laying and fertilization compared with *Mst89B* KDs, this is more likely explainable by stochastic variation caused by differences between individuals in effectiveness of the KD. Given that the seminal vesicle of *Mst89B* and *CG31287* KD males contained large amounts of sperm (Figure 3A), we tested whether egg hatchability or larval viability were impaired. We found that females mated to *Mst89B* and *CG31287* KD males hatched a significantly lower proportion of eggs than controls (6.7% and 33.1% respectively compared with 92.1% for controls; Chi-squared test, controls vs. gene KDs: *P* < 0.001) (Figure 3B).

However, we found no significant differences in larva to pupa and pupa to adult viability (Kruskal-Wallis: larva to pupa, H = 2.59; *p* = 0.274, and pupa to adult, H = 4.49; *p* = 0.106) (Table 1).

### 3.2. Knockdown of Mst89B and CG31287 Does Not Affect Sperm Transfer but Impairs Long-Term Sperm Storage

**Figure 4:**
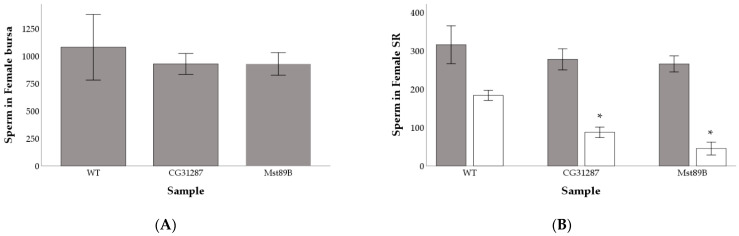
Average number of sperm cells in the female bursa 30 min after the end of copulation (**A**) and average number of sperm found in the female seminal receptacle (SR) at 30 min (grey bars) and 24 h (white bars) after mating (**B**). Asterisks (*) above bars denote significant differences between the samples and the wild-type control. Error bars are 2 standard errors of the mean.

We investigated whether the ability of *Mst89B* and CG31287 KD males to transfer and store sperm was hindered (Figure S3). We found that *Mst89B* and *CG31287* KD males transferred large amounts of sperm to the female bursa that were comparable to control males (F_2,26_ = 0.907; *p* = 0.416) (Figure 4A). Additionally, females mated to *Mst89B* and *CG31287* KD males had a similar amount of sperm in their seminal receptacle to females mated to control males at 30 min AEC (F_2,32_ = 2.257; *p* = 0.121) (Figure 4B). However, at 24 h AEC, the amount of sperm in the SR of females mated to *Mst89B* and *CG31287* KD males was significantly different among samples, with significantly reduced amounts of sperm in females mated to KDs relative to those mated to control males (F_2,32_ = 96.820; *p* < 0.001; pairwise Scheffe’s post-hoc tests comparisons wild-type vs. KDs q < 0.001) (Figure 3B). Compared with the control females, the SR of females mated to *CG31287* and *Mst89B* KD males showed approximately a 52% and 75% decrease in the amount of sperm stored at 24 h AEC, respectively.

## 4. Discussion

Given that the majority of the *Drosophila melanogaster* genome is made of non-coding sequences that might serve as putative regulatory elements [32], it becomes imperative to understand how perturbations of gene expression can affect traits such as male fecundity. Additionally, broad rather than detailed phenotypic characterization of fecundity itself, combined with the polygenic nature of the trait, have made it difficult to identify genes influencing sperm function and fecundity [11]. A male’s reproductive fitness can be drastically reduced due to challenges sperm cells face after transfer to the female and prior to fertilization. For example, subtle problems in the sperm itself, or other components of the ejaculate composition, can impair the ability of sperm to remain in female storage, affect its localization inside the female reproductive tract, incapacitate movement within and between storage organs, and influence the sperm fertilization capacity [3,4,7,33,34,35,36,37,38,39]. Here, we focused our genetic survey to a specific locus (89B) previously identified as a hub for genes with roles in male fecundity [25,26] and have used RNAi to test the effect of single gene perturbation on different aspects of male fecundity. We lack null mutants that could be used to establish a better relationship between gene and phenotype, but our primary interest is in assessing the effect of modifications in gene expression, rather than the effect of complete gene loss, in post-mating success phenotypes. Nevertheless, RNAi can give false positives due to off-target effects by base-pairing with unintended mRNAs [40]. In what follows, we discuss our findings for the germline KD of 5 genes affecting fertility. Some of our phenotypic characterizations confirm previous results but others are novel. To address the possibility that some of the observed phenotypes could be off-target artifacts, we use the Updated Targets of RNAi Reagents website (UP-TORR) (https://www.flyrnai.org/up-torr/About.jsp) [41] for in silico examination of sequence similarities to other genome targets. However, in the absence of independent RNAi non-overlapping gene target experimental controls or RNAi rescue experiments, a caveat remains as we cannot fully rule out possible off-target effects.

KD of *CSN5* and *bor* rendered males fully sterile. These KDs appear to have normal sperm elongation, but their seminal vesicles are devoid of sperm. We are not able to see any break down before spermatid elongation, but subtle pre-elongation defects might be missed in our assay, as Cyst cell-RNAi against *CSN5* (*COP9 signalosome subunit 5*) has been claimed to cause a weak defect in germline enclosure and differentiation [42]. We found no in silico evidence for off-target effects of our *CSN5* RNAi construct. In *Caenorhabditis elegans*, KD of *atad-3*, the ortholog of *bor* (*belphegor*) has also been shown to cause sterility despite the gonadal morphology of the animals appearing normal [43]. In *Drosophila*, manipulation of *bor* expression in larva has been shown to affect mitochondrial morphology and function and to increase autophagy [44]. However, our detection of a role in adult male sperm production and fertility in *Drosophila* is novel. The *bor* KD construct has an off-target effect to *CG45063*, a gene of unknown function with low testes expression. *Manf* (*Mesencephalic astrocyte-derived neurotrophic factor)* is known to play a role in neuronal development and function [45,46], and here we have identified a new function of this gene as it is required for production of mature sperm. Lindström et al. [47] suggested that, based on the localization of Manf in the endoplasmic reticulum (ER), it could partake in ER-mitochondrial crosstalk, and disturbances in Manf protein levels could affect protein transport to the mitochondria. In *Drosophila*, post meiosis II (i.e., pre-elongation), giant mitochondria fuse and pack into a structure called the nebenkern, which differentiates into major and minor mitochondrial derivates that run along the entire length of an elongated spermatid [48]. Mutant flies with small mitochondrial derivates produce spermatids with severe elongation defects, and giant mitochondria are required to provide structural support to elongating spermatids [49]. However, *Manf* KDs appear to have normal spermatids, were able to produce few mature sperm cells, and had low fecundity. While we cannot identify defective spermatid elongation, it is possible that mitochondrial function may be somehow impacted in *Manf* KDs, affecting spermatids maturation. The *Manf* RNAi construct can potentially exert its effect through two off-target genes. One is *Sulfated* (*Sulf1*), an enzyme with low testes expression that regulates Wingless (Wg) and Hedgehog (Hh) signaling during development (http://flybase.org/). More importantly, the other off-target is *mei-P26*, which is involved in germline differentiation and spermatogenesis, and thus an off-target effect of *mei-P26* might contribute to our detected phenotype. While this remains to be tested, phase contrast imaging of *mei-P26* mutants show a very different testes phenotype than the one we report here for the *Manf* KD, with over-proliferation of spermatogonial cysts at the testes apical end [50].

We show that the germline KD of *Mst89B* drastically affects male fecundity by impairing the ability of sperm to properly fertilize eggs once in female storage. We did not detect any off-target effect for the *Mst89B* RNAi construct. Previously, we found that the KD of *Mst89B* with the *Act5C*-Gal4 driver did not affect fecundity but led to a decrease in second male paternity success despite no breakdown of the second male advantage (i.e., P2 remained higher than 0.5) [26]. The *Act5C* promoter has been shown to be ineffective in driving transgenes expression in the germline [27], so it is possible that our previous result was caused by perturbation of the gene expression in somatic cells. It is also possible that the *Act5C*-Gal4 driver induced a lesser germ-line perturbation, given the weaker expression of the transgene in the germline, than the *bam*-Gal4 driver. Similarly, KD of *CG31287* with the *Act5C*-Gal4 driver did not affect male fecundity in non-competitive or competitive assays [26] but here, when KD with *bam*-Gal4, resulted in a similar phenotype as the *Mst89B* KD. The *CG31287* RNAi construct had no detectable off-target effects.

Functionally, not a lot is known about *Mst89B* or *CG31287*, except that *Mst89B* is a cytoplasmic protein expressed during spermatogenesis only in germline-derived cells [51]. Several genes required for the normal functioning of sperm have been previously identified using single-gene perturbation assays. For example, *Dnah3*, is required to maintain sperm motility, without which *Dnah3* mutants are infertile [33]. Loss of *Sdic* leads to an inability to displace resident sperm from the storage organs leading to fewer second-male progeny [8,52], even though it does not result in morphological defects in sperm nor does it impair motility or the ability of males to sire progeny [8]. In a similar fashion to the KD of *CG31287* and *Mst89B*, *Pkd2* and *shps* mutants produce and transfer normal amounts of sperm to the female bursa, but significantly fewer eggs are fertilized [36,53]. However, in the case of *Pkd2* and *shps*, mutant sperm are unable to enter storage. *Pkd2* mutant sperm form abnormal flagellar waveforms, which inhibit their entry into the SR [34,35]. Mutation of *shps* also affects sperm motion parameters, but it is unclear whether the defective motion impedes the sperm’s ability to enter storage [36].

In addition to low hatchability, we found that females mated to *CG31287* and *Mst89B* KD males had fewer sperm in their storage organs 24 h AEC. Sperm release from the storage organs is known to be mediated by several seminal fluid proteins, with a number of proteins interacting with sex peptide, a seminal fluid protein bound to sperm that affects sperm release [6,18,54,55,56,57,58]. Only the loss-of-function mutation of *Acp29AB* results in faster depletion of sperm from the storage organs, but it does not affect hatchability or fecundity [55]. There is at least one known gene that is required for the normal release of sperm from storage and egg fertilization. Like the KDs of *CG31287* and *Mst89B*, mutation of the gene *wasted* (*wst*) does not affect sperm transfer or storage, but compared with controls, 92% of the stored sperm is lost from the seminal receptacle within 24 h after mating [7]. Furthermore, fewer sperm from *wst* mutants enter the egg for fertilization compared with sperm from controls, and the majority that do enter the egg are unable to initiate mitotic divisions, resulting in reduced hatchability [7]. Given the similarity of phenotypes observed between *wst* mutants and our KDs of *CG31287* and *Mst89B*, it is possible that these genes are functionally related. The development of stable transgenic lines using gene editing and the incorporation of fluorescent markers into the mutants should facilitate further functional characterization of these two genes.

## Figures and Tables

**Table 1:** Average proportion of larva to pupa (P/L) and pupa to adult (A/P) viability for wild-type controls and knockdowns (KD) of genes Mst89B and CG31287.

Treatment	N	P/L	A/P
Control	96	0.89	0.93
*CG31287*	86	0.84	0.99
*Mst89B*	44	0.94	0.97

## Data Availability

The data presented in this study are openly available in Dryad (https://datadryad.org) at doi:10.5061/dryad.jh9w0vt9w.

## Supplementary Materials

The following are available online at https://www.mdpi.com/2073-4409/10/2/289/s1, Table S1: Genes located in 89B and their expression level in testes, Table S2: Stocks used to generate KDs of genes with high expression in testes and primers information, Figure S1: Test of expression level of candidate reference genes RpS18, eEF1α1 and αTub84B in the male reproductive tract of KD males, Figure S2: Expression of genes in KDs relative to control wild type (wt) males, Figure S3: Microscopy images of sperm in the male seminal vesicle (SV) and the female storage organs.
