# Supplementary material for: Expression of Mst89B and CG31287 is Needed for Effective Sperm Storage and Egg Fertilization in Drosophila
Source: Cells. 2021 Feb 1;10(2):289. doi: 10.3390/cells10020289 (PMC7912738; doi:10.3390/cells10020289)

Supplementary Material

Table S1. Genes located in 89B and their expression level in testes based on RNA sequencing (RNAseq) and microarray (MicroA) data. Genes selected for testing are bolded. Yellow to red is used for high, very high and extremely high expression respectively (Flybase).

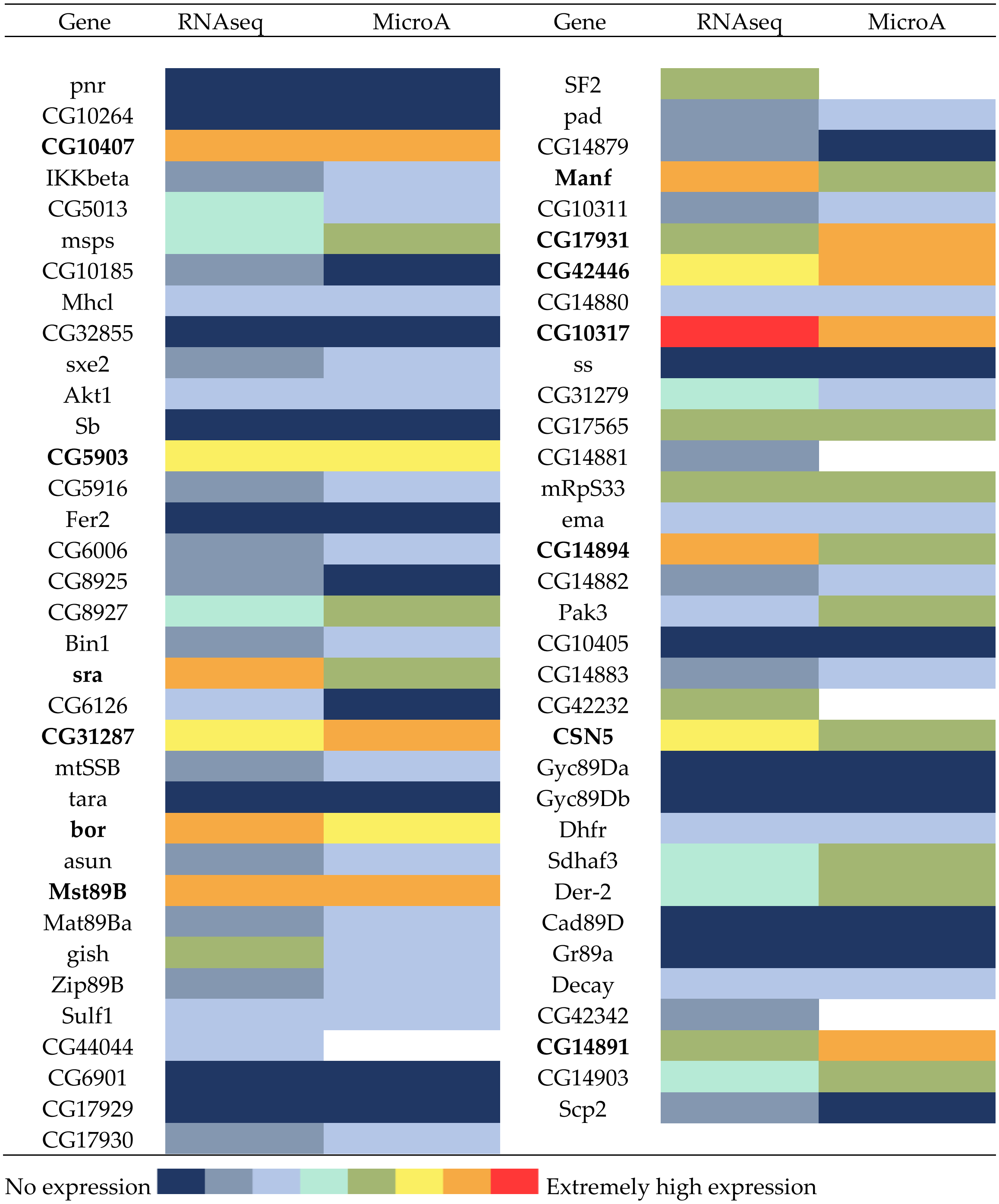

Table S2. Stocks used to generate knockdowns of genes with high expression in testes and primers information. The stocks were purchased from the Vienna Drosophila Resource Center (VDRC) and Bloomington Drosophila Stock Center (BDSC). Primer sequences, amplicon size (AS), and primer efficiency (E) of all the candidate genes and reference genes tested. NA= Non-available or Non-applicable.

Gene	Stock	Primer Sequence	AS	E (%)
CG42446	NA	NA	NA	NA
CG5903	66933 (BDSC)	F: TGTCGGCCATTACGTGGAAA<br>R: GAGTTCTGGGGCTCGTTCAG	75	91.08
CG6072 (sra)	107573 (VDRC)	F: GACGTGGATAGCGACACAGA<br>R: TGGATGTTGGTCACGATGAT	83	96.76
CG6815 (bor)	110208 (VDRC)	F: CGGTTCAAACTGGACACCTT<br>R: ATTTCTCGACCCGACATACC	83	113.44
CG6864 (Mst89B)	102666 (VDRC)	F: CCACGCACTTCTGCACTTT<br>R: CAACTGTCTTCAGCGGGTTC	81	98.5
CG7013 (Manf)	100814 (VDRC)	F: AAACCCCTCAGTTGGTCCAT<br>R: CATAGCGAAGGTCGCAGATT	85	92.53
CG10317	105916 (VDRC)	F: GCCACTCAAAAGAACCAGGA<br>R: CCGTCCTCTGGTTTTCCTCT	79	112.55
CG10407	104526 (VDRC)	F: CTGGACAACAGCCAAACCTC<br>R: GTGTCTAGGTCGGGTGCATT	90	102.5
CG14894	103701 (VDRC)	F: AACGGGAGAAGGATCTCAGC<br>R: ACTCGTTGCCCTCAACTTTC	81	116.65
CG31287	103437 (VDRC)	F: AAAGTGCACCATGCTTAGGG<br>R: TAACTGCTTTCTCCGCCTGT	77	99.7
CG14884 (CSN5)	62970 (BDSC)	F: AAGGTGTGTCTGGGTGCATT<br>R: CAGCGGGATGGTCTGATACT	87	111.64
CG14891	102118 (VDRC)	F: TCTGTCTCGTCTCCACACCT<br>R: TCGACGTAAGCAGCACGTTA	80	111.69
CG17931	100894 (VDRC)	F: AATAGCAAGTGCCACCCAAG<br>R: ATCGTACAGCAGCACTGCAA	76	116.89
CG8900 (RPS18)	NA	F: GGTGACCATCATCTCGAACC<br>R: GCCATCGATGATGTCCTTCT	79	107.22
CG8280 (eEF1α1)	NA	F: TGGAGACTGGTGTGCTGAAG<br>R: CATCTCCACGGACTTGACCT	83	97.12
CG1913 (αTub84B)[†]	NA	F: TGTCGCGTGTGAAACACTTC<br>R: AGCAGGCGTTTCCAATCTG	96	103.85

[†]Primer sequences were obtained from Ponton et al. (2000).

Ponton, Fleur, et al. "Evaluation of potential reference genes for reverse transcription-qPCR studies of physiological responses in Drosophila melanogaster." Journal of insect physiology 57.6 (2011): 840-850.

Figure S1: Test of expression level of candidate reference genes *RpS18*, *eEF1α1* and *αTub84B* in the male reproductive tract of knockdown males. Individual gene knockdowns were driven by *bam*-Gal4 for genes CG10317, CG17931, and CG14894.

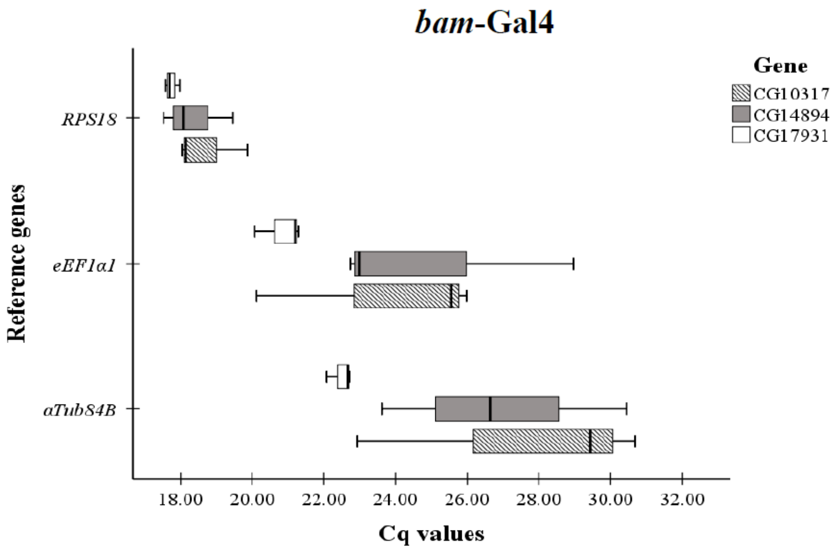

Figure S2: Expression of genes in knockdowns (KDs) relative to wild type (wt) control males. P-values from all one-tailed paired t-test comparisons are lower than 0.001, except when shown.

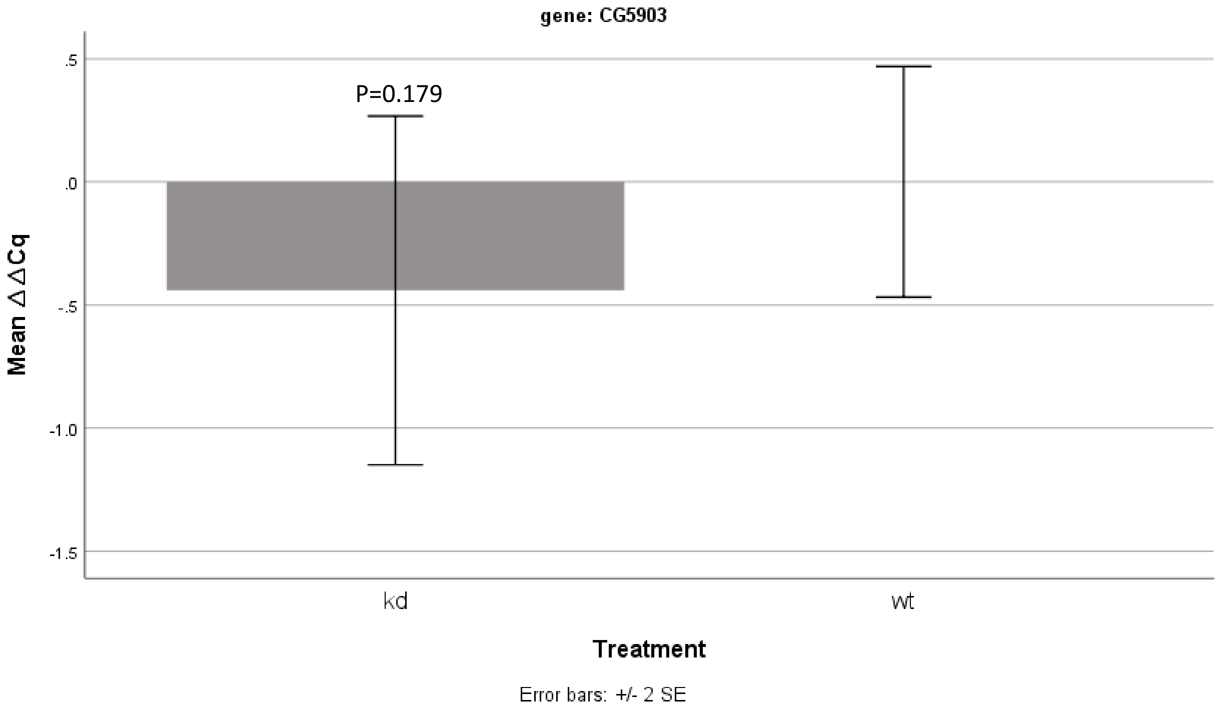

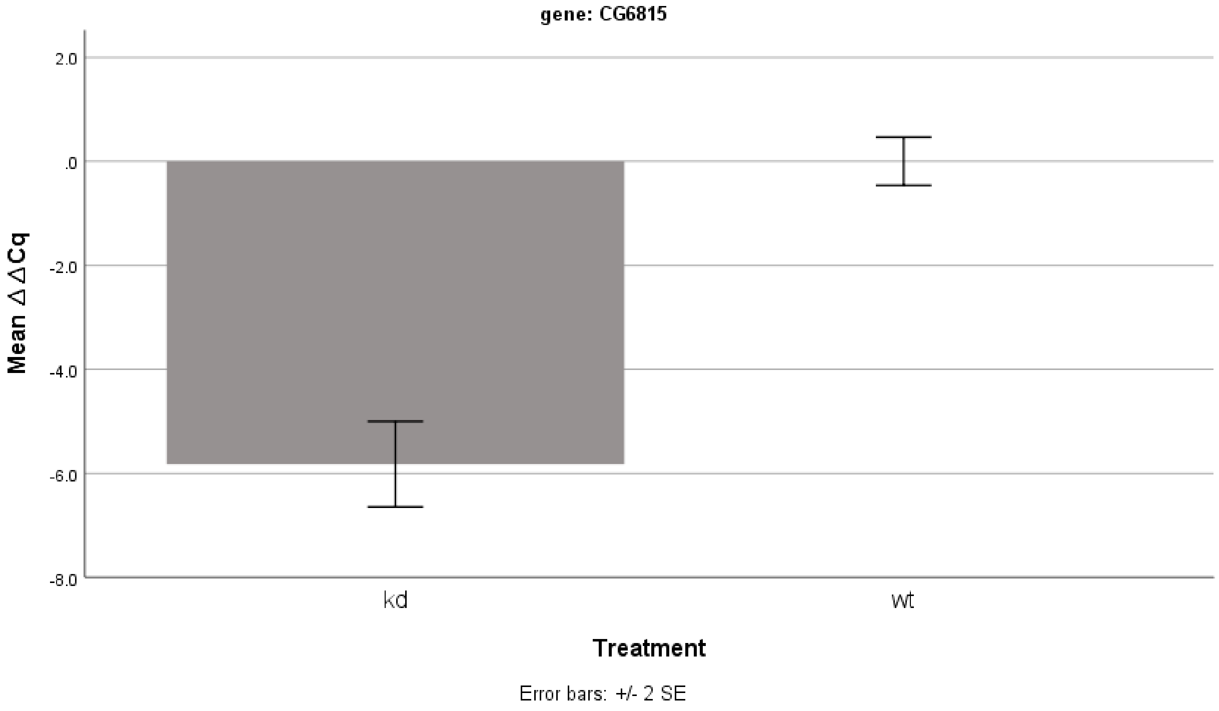

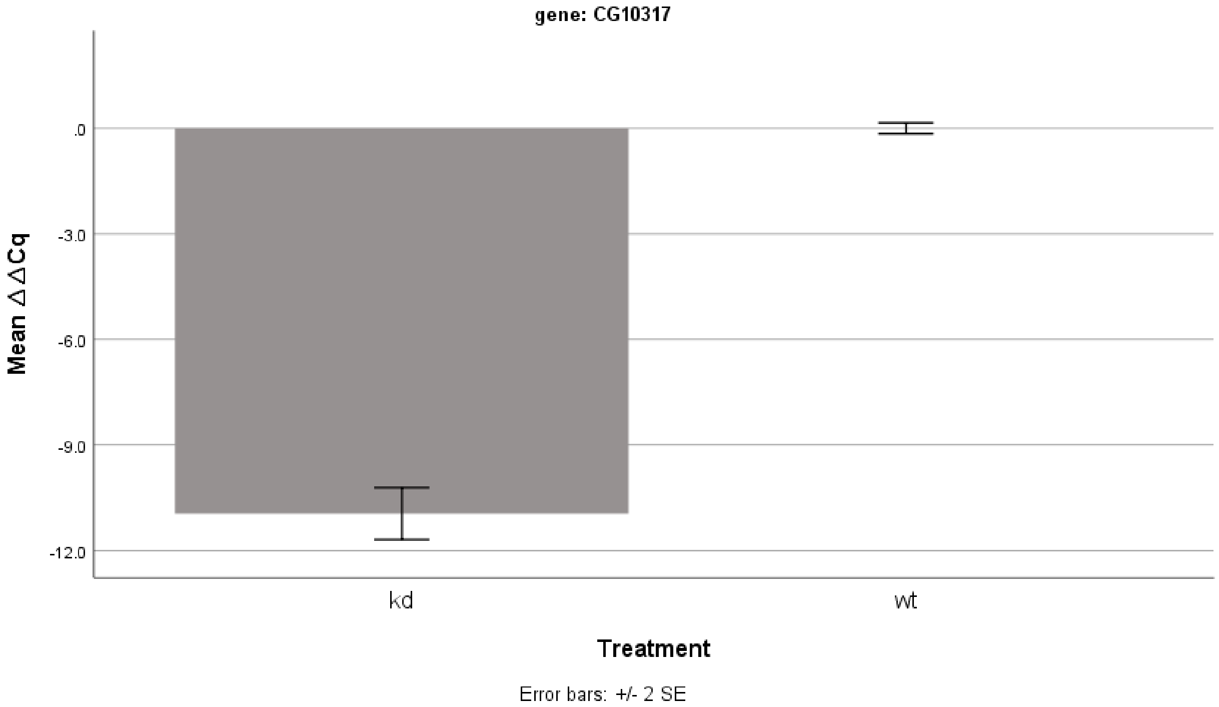
gene: CG10317
Mean ΔΔCq
.0
-3.0
-6.0
-9.0
-12.0
kd
wt
Treatment
Error bars: +/- 2 SE

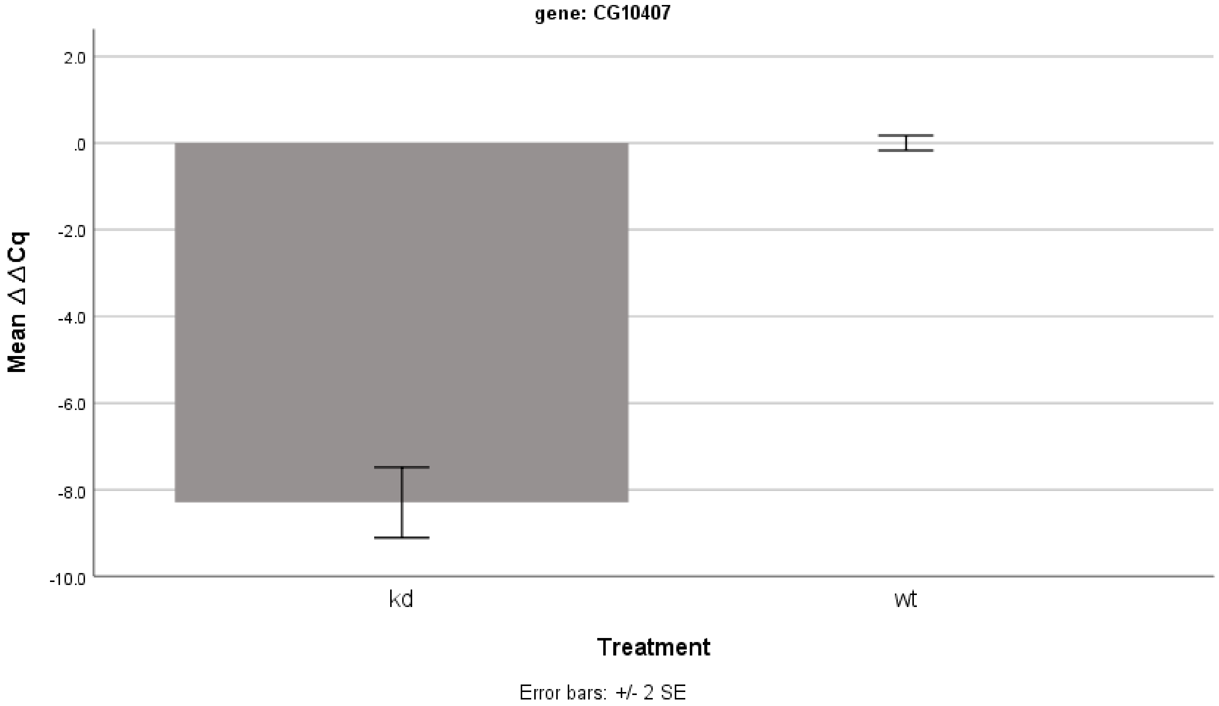
gene: CG10407
Mean ΔΔCq
2.0
.0
-2.0
-4.0
-6.0
-8.0
-10.0
kd
wt
Treatment
Error bars: +/- 2 SE

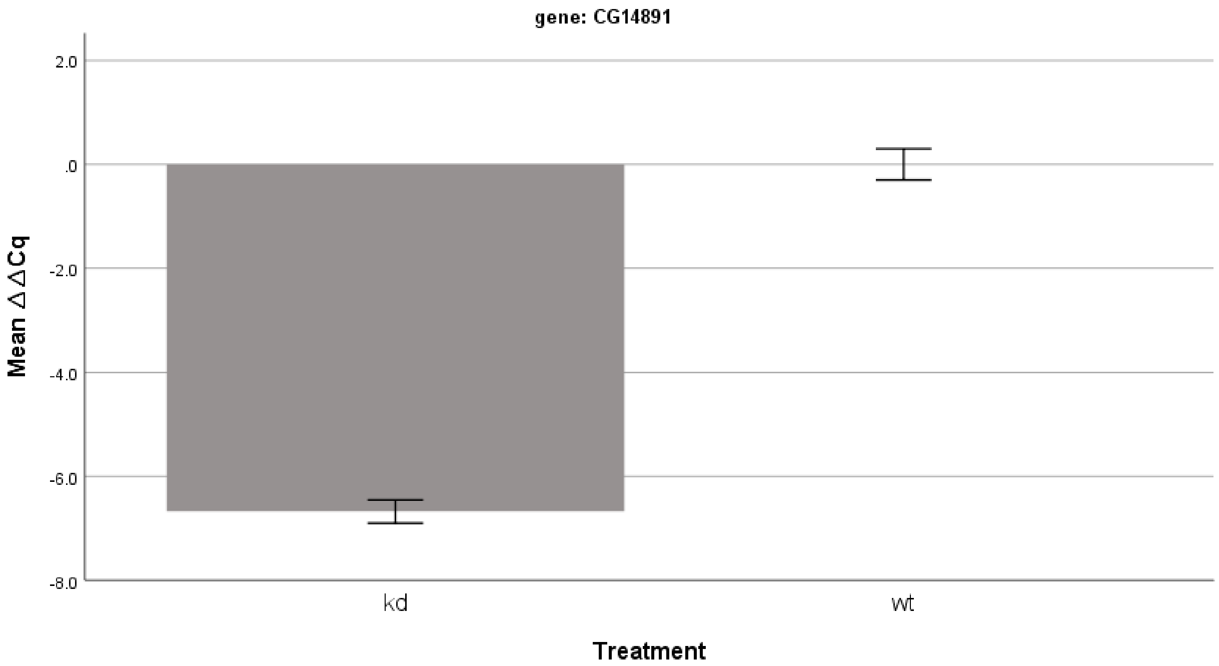

Error bars: +/- 2 SE

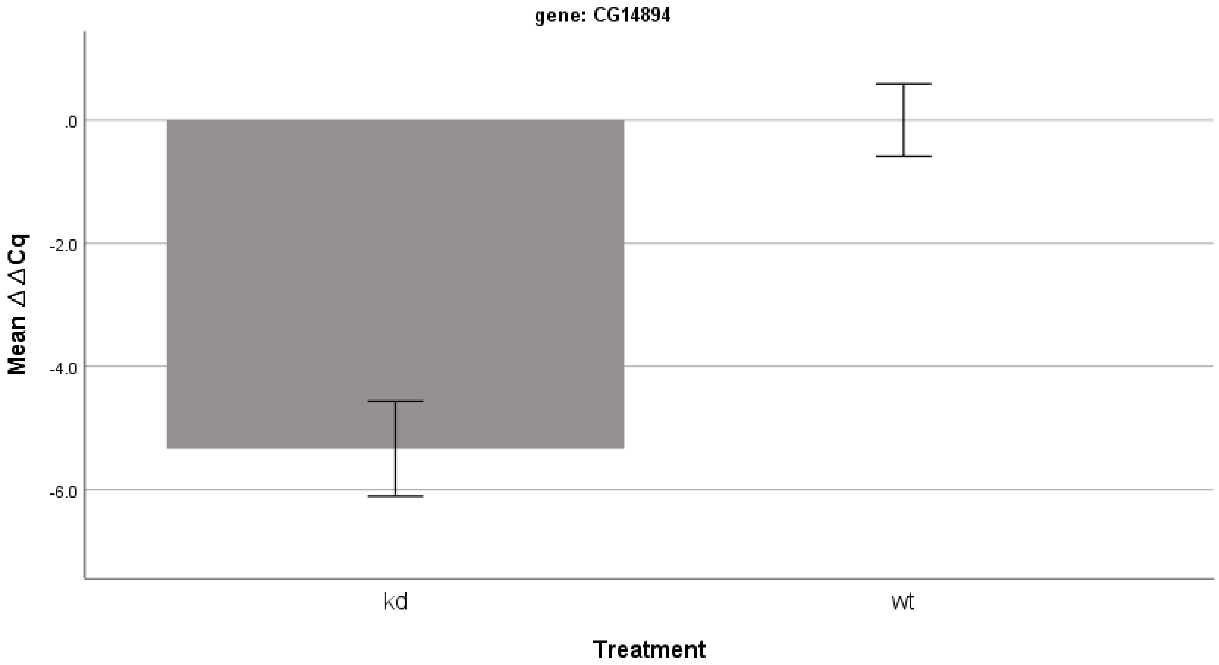

Error bars: +/- 2 SE

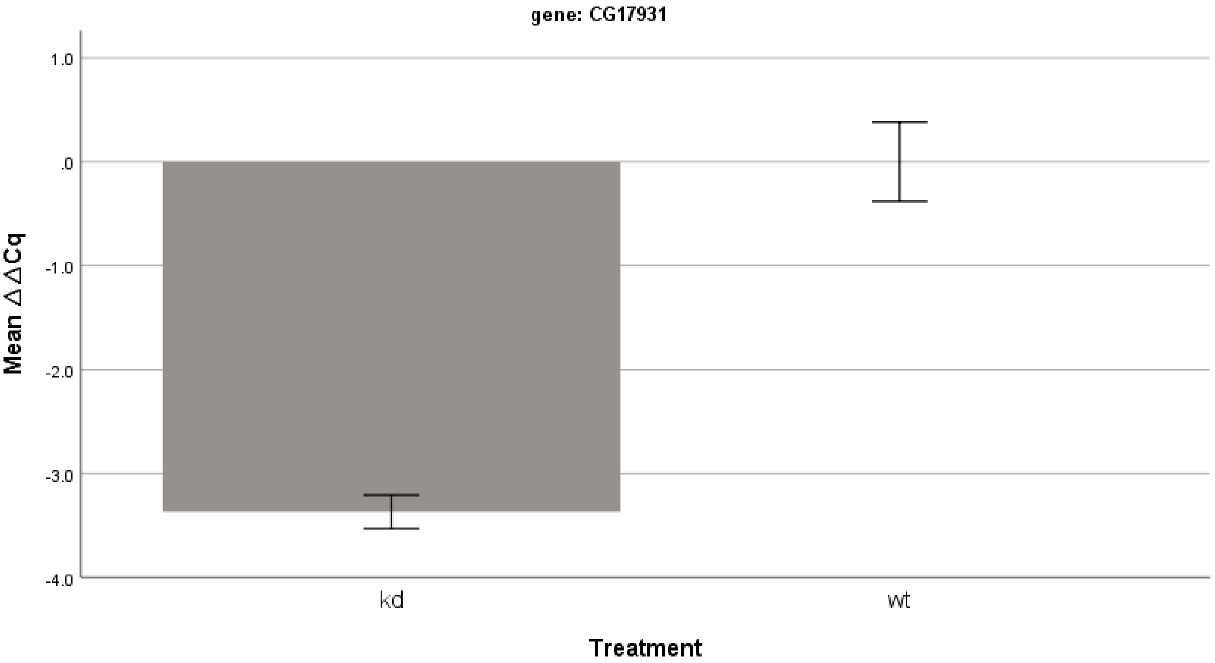

Error bars: +/- 2 SE

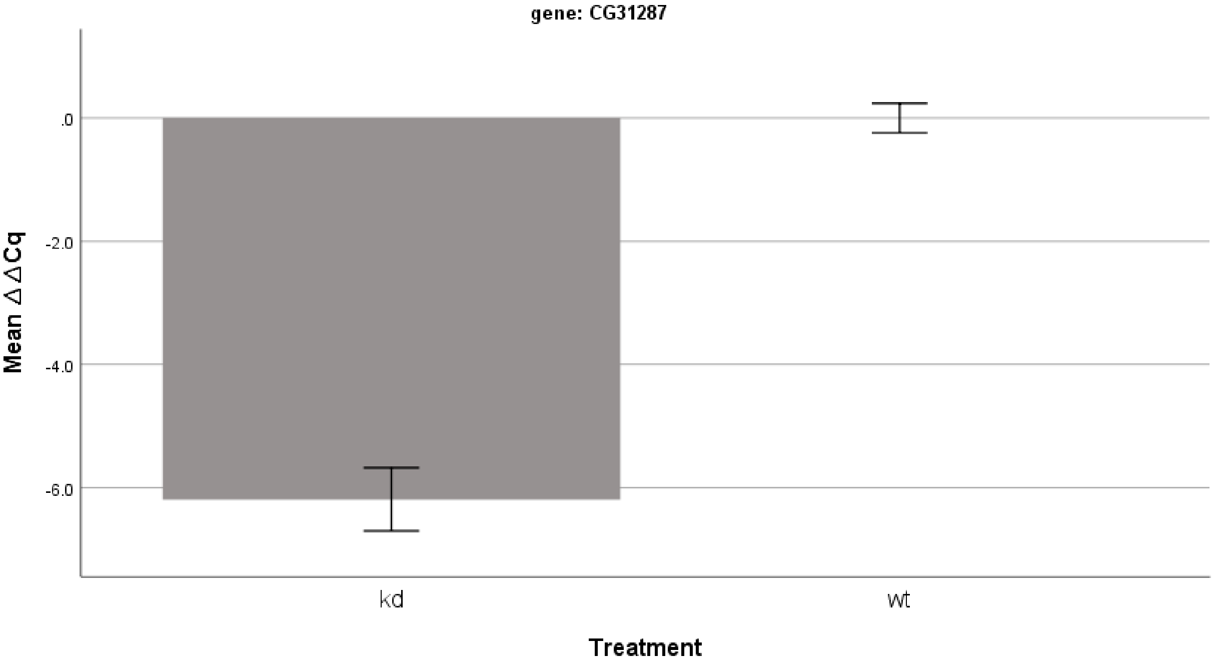

Error bars: +/- 2 SE

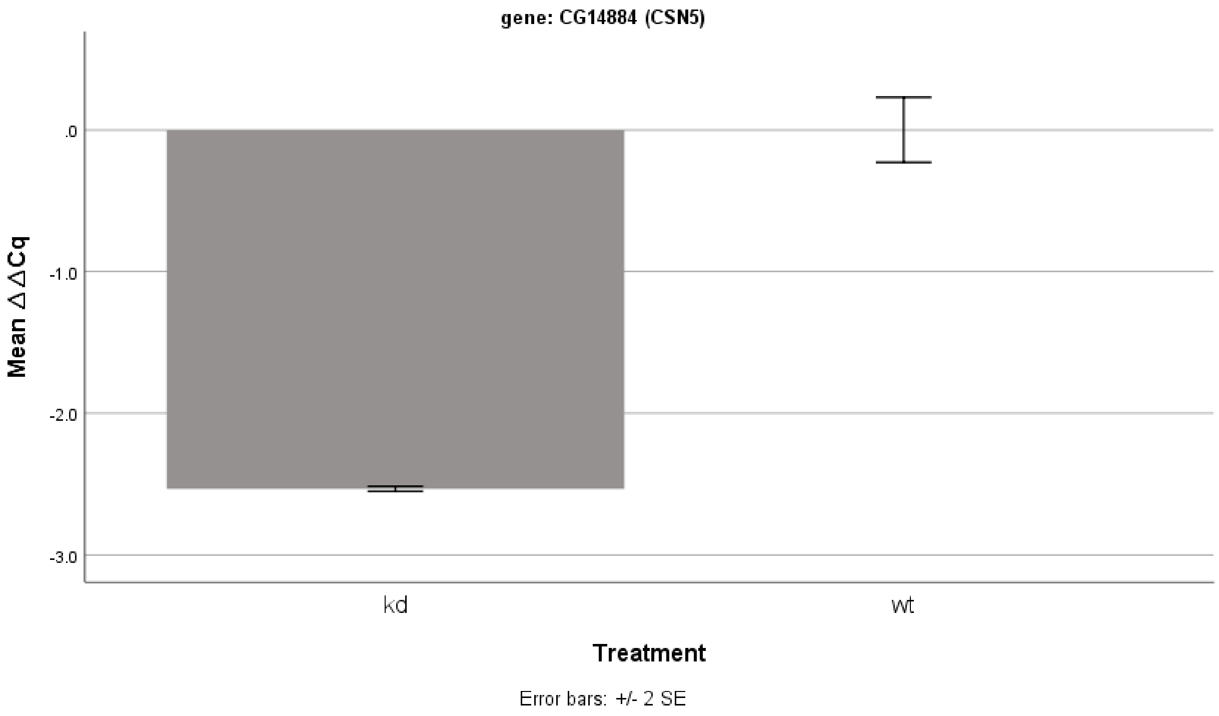
gene: CG14884 (CSN5)
Mean ∆∆Cq
.0
-1.0
-2.0
-3.0
kd
wt
Treatment
Error bars: +/- 2 SE

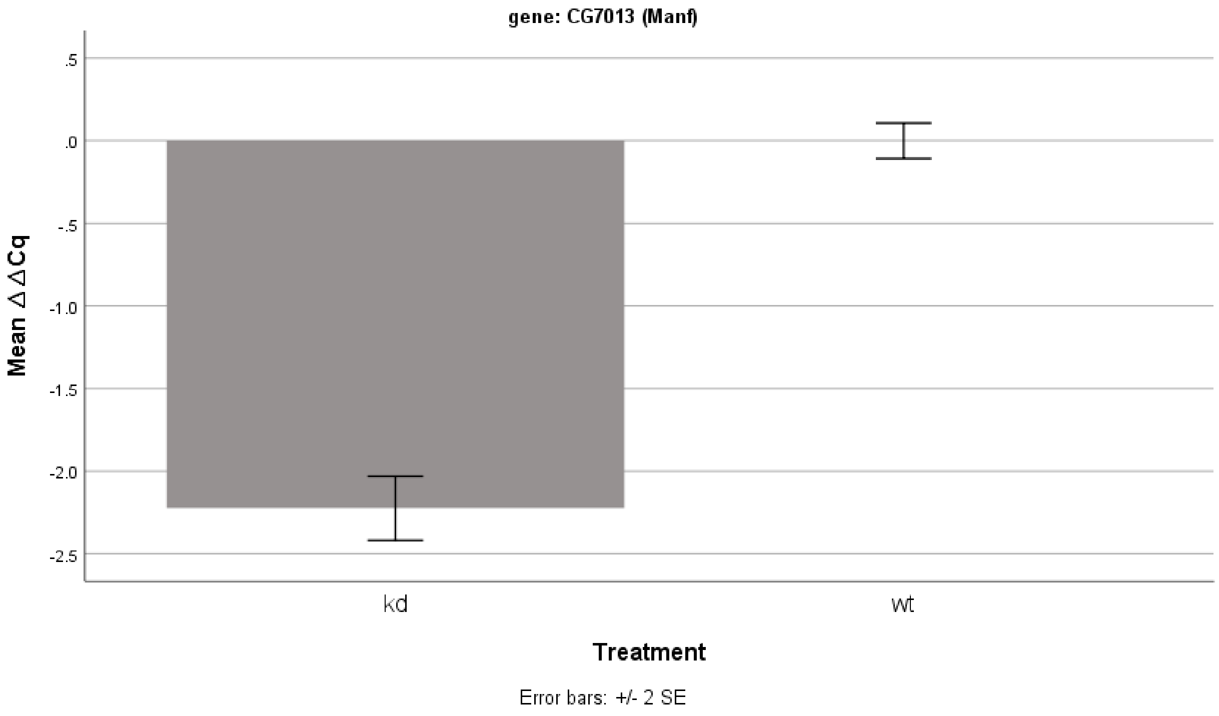
gene: CG7013 (Manf)
Mean ∆∆Cq
.5
.0
-.5
-1.0
-1.5
-2.0
-2.5
kd
wt
Treatment
Error bars: +/- 2 SE

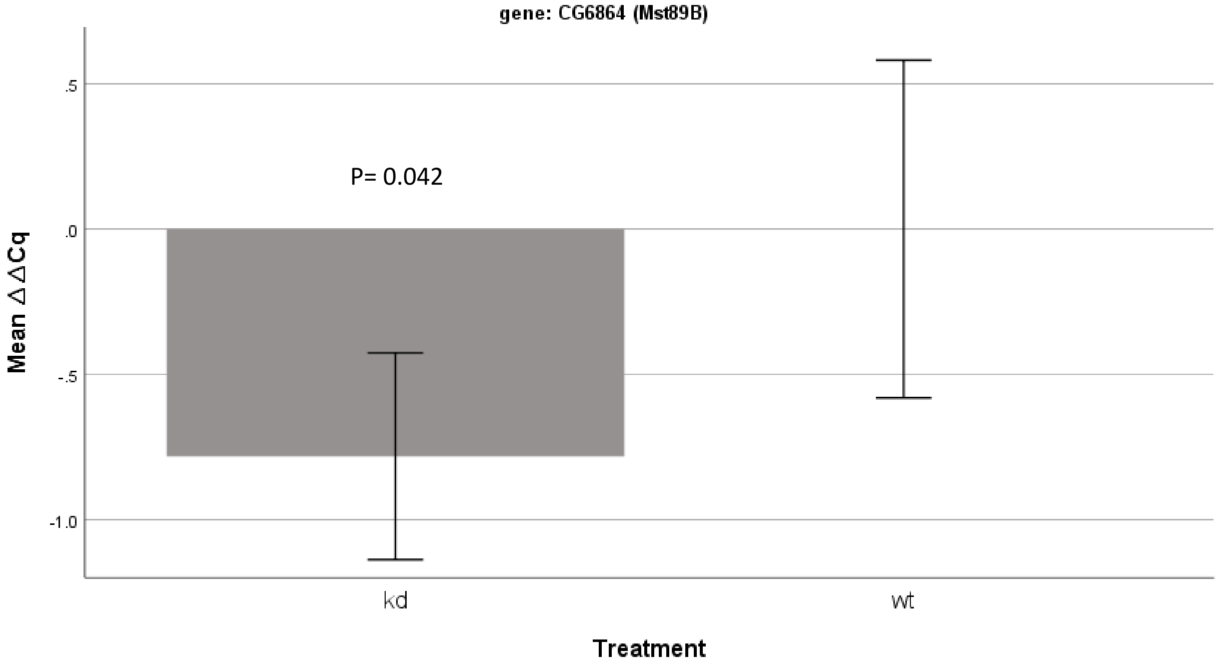

Error bars: +/- 2 SE

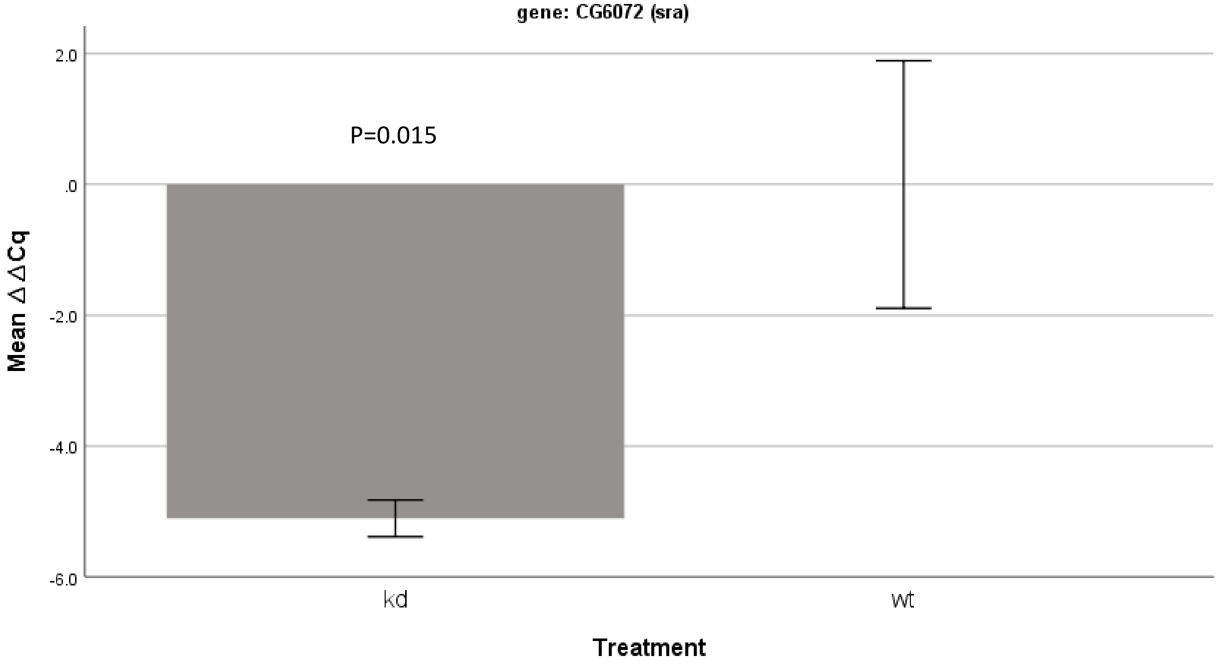

Error bars: +/- 2 SE

Figure S3: Microscopy images of sperm in the male seminal vesicle (SV) and the female storage organs. Sperm cells are seen as needle-like structures. The female images are for the bursa (Bu) and a section of the seminal receptacle (SR).

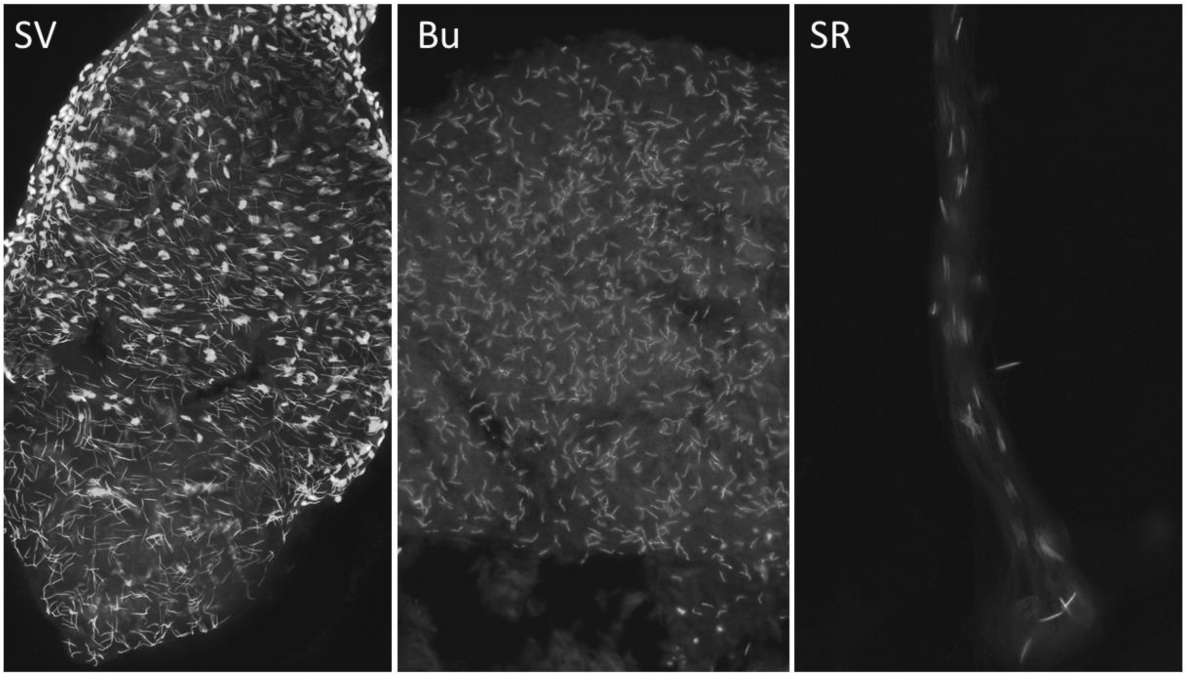

Supplement: Supplementary file 1 [file cells-10-00289-s001.pdf]
